# Geno- and phenotypic characteristics of a transfected *Babesia bovis* 6-Cys*-E* knockout clonal line

**DOI:** 10.1186/s13071-017-2143-3

**Published:** 2017-05-02

**Authors:** Heba F. Alzan, Marta G. Silva, William C. Davis, David R. Herndon, David A. Schneider, Carlos E. Suarez

**Affiliations:** 1 Department of Veterinary Microbiology and Pathology, College of Veterinary Medicine, Washington State University, Pullman, WA USA; 2 Parasitology and Animal Diseases Department, National Research Center, Dokki, Giza, Egypt; 3 Animal Disease Research Unit, United States Department of Agriculture - Agricultural Research Service, Pullman, WA USA

**Keywords:** *B. bovis* transfection, 6-Cys *E* gene, Cell sorting, Transfected clonal line

## Abstract

**Background:**

*Babesia bovis* is an intra-erythrocytic tick-transmitted apicomplexan protozoan parasite. It has a complex lifestyle including asexual replication in the mammalian host and sexual replication occurring in the midgut of host tick vector, typically, *Rhipicephalus microplus*. Previous evidence showed that certain *B. bovis* genes, including members of 6-Cys gene family, are differentially expressed during tick and mammalian stages of the parasite’s life cycle. Moreover, the 6-Cys *E* gene is differentially expressed in the T3Bo strain of *B. bovis* tick stages, and anti 6-Cys E antibodies were shown to be able to inhibit in vitro growth of the phenotypically distinct *B. bovis* Mo7clonal line.

**Methods:**

In this study, the 6-Cys *E* gene of *B. bovis* T3Bo strain was disrupted by transfection using a plasmid containing 6-Cys gene *E* 5′ and 3′ regions to guide homologous recombination, and the *egfp-bsd* fusion gene under control of a *ef-1α* promoter, yielding a *B. bovis* clonal line designated 6-Cys EKO-*cln*. Full genome sequencing of 6-Cys EKO-*cln* parasites was performed and in vitro inhibition assays using anti 6-Cys E antibodies.

**Results:**

Full genome sequencing of 6-Cys EKO-*cln B. bovis* demonstrated single insertion of *egfp-bsd* gene that disrupts the integrity of 6-Cys gene *E*. Undistinguishable growth rate of 6-Cys EKO-cln line compared to wild-type 6-Cys E intact T3Bo *B. bovis* strain in in vitro cultures indicates that expression of gene 6-Cys E is not essential for blood stage replication in this strain. In vitro inhibition assays confirmed the ability of anti-6 Cys E antibodies to inhibit the growth of the wild-type Mo7 and T3Bo *B. bovis* parasites, but no significant inhibition was found for 6-Cys EKO-*cln* line parasites.

**Conclusions:**

Overall, the data suggest that the anti-6 Cys E antibody neutralising effect on the wild type strains is likely due to mechanical hindrance, or cross-reactivity, rather than due to functional requirements of 6-Cys gene *E* product for survival and development of the erythrocyte stages. Further investigation is underway to determine if the 6-Cys E protein is required for replication and sexual stage development of *B. bovis* during tick stages.

**Electronic supplementary material:**

The online version of this article (doi:10.1186/s13071-017-2143-3) contains supplementary material, which is available to authorized users.

## Background

*Babesia bovis* is an apicomplexan tick-borne parasite, mainly transmitted by *Riphicephalus microplus,* responsible for acute disease in bovines. The disease causes important economic loses in endemic areas worldwide, and improved methods of control are needed. A common method used to prevent acute babesiosis in endemic areas, such as Australia, Argentina and Mexico, is the use of *B. bovis* attenuated vaccines, which have multiple limitations [1]. Subunit vaccines able to elicit sterile immunity or prevent acute babesiosis would be ideal prevention tools, but they remain unavailable. Additional tools for enhanced control include vaccines designed to block transmission of the parasites by the tick vector. Development of such vaccines requires the identification of relevant antigens expressed in the tick stages of the parasites. Recent work resulted in the identification of candidate antigens that can be used for the developing of *Babesia* transmission blocking vaccines [2], including the members of the 6-Cys gene family. The apicomplexan 6-Cys gene family was originally identified in the *Babesia*-related *Plasmodium* parasites [3–5]. Members of the 6-Cys family are defined by a unique arrangement of 6-Cysteine residues, although other alternative arrangements involving 4, 5 and seven cysteine residues are also possible [2]. The members of the 6-Cys family contain signal peptides and are thus likely expressed on the surface of the parasites [2]. Interestingly, *Plasmodium* 6-Cys proteins such as pfs230 and s48/45 are expressed in sexual stages of the parasite, occurring exclusively in the midgut of the mosquito vector, whereas other *Plasmodium* 6-Cys proteins are differentially expressed in the sporozoite and or merozoite stages [6]. *Plasmodium* 6-Cys proteins are known to play important roles in the sexual stage forms and are required in oocyte formation, and are strong candidates for the development of transmission-blocking vaccines [6, 7]. In addition, some *Plasmodium* 6-Cys proteins such as 6-Cys protein P12 and P41 are also known to be expressed in erythrocyte stages of the parasite [8]. Similar to *Plasmodium* parasites, *B. bovis* encodes for 6-Cys proteins [9]. The *B. bovis* 6-Cys gene family was originally described as containing six genes termed 6-Cys A-F, but, further searches of the genome revealed the presence of four additional genes (6-Cys G-J), in this family [2]. Currently, the roles of the 6-Cys gene family members in survival during the tick and mammalian stage of the life-cycle are unknown. The gene 6-Cys E was found transcribed only in tick stages, but not in the blood stages of parasites of the T3Bo strain [2]. In addition, previous studies performed on the biologically cloned *B. bovis* strain Mo7 suggested that the 6-Cys E protein might be a suitable candidate for a subunit vaccine because it was found to be expressed on the surface of the cloned *B. bovis* Mo7 strain, while developing in the blood stage, [9] and contained neutralization sensitive epitopes. We were interested in further analysing the biological significance of this previous finding and into determining whether expression of 6-Cys E is essential for the survival of the blood stage of the life-cycle of *B. bovis*. Importantly, if 6-Cys *E* mutants are viable and able to develop in in vitro cultures, it would provide an essential tool for further testing of the possible functional role of the 6-Cys *E* gene in erythrocyte and tick stages of the life cycle of *B. bovis*.

The focus of the present study was to determine whether expression of the 6-Cys gene *E* is needed for the replication of the blood stage of the parasite in vitro. Here, we describe the production of a *B. bovis* 6-Cys *E* knockout (KO) clonal line derived from the T3Bo strain of *B. bovis* using transfection methods [9–13] that can be applied for future gene functional analysis. The *B. bovis* T3Bo line was selected for this study because recent unpublished evidence suggests that the phenotypically distinct Mo7 strain, which was used in previous studies, is not likely transmissible by ticks, and therefore not suited to perform further tick transmission studies required to prove the role of 6-Cys proteins in transmission.

We also compared the ability of anti-6-Cys E antibodies to inhibit the in vitro growth of KO and wild-type *B. bovis* parasites. Collectively, the results indicated that the gene 6-Cys *E* is not essential for the development of erythrocyte stages of *B. bovis*. Although the results are not supportive of using the 6-Cys E as a blood stage vaccine component, they suggest that this protein could still be a candidate for developing transmission blocking vaccines against *B. bovis*.

## Methods

### Parasites

The Texas (Tx) T3Bo [14] and Mo7 strains of *B. bovis* were grown in long-term microaerophilic stationary phase culture as previously described [10, 11]. Both strains*,* Mo7 and T3Bo, of *B. bovis* [10, 12] are maintained as a cryopreserved stabilate in liquid nitrogen when not in use [13].

### Construction of the transfection plasmid *p6-Cys-EKO*

**Fig. 1:**
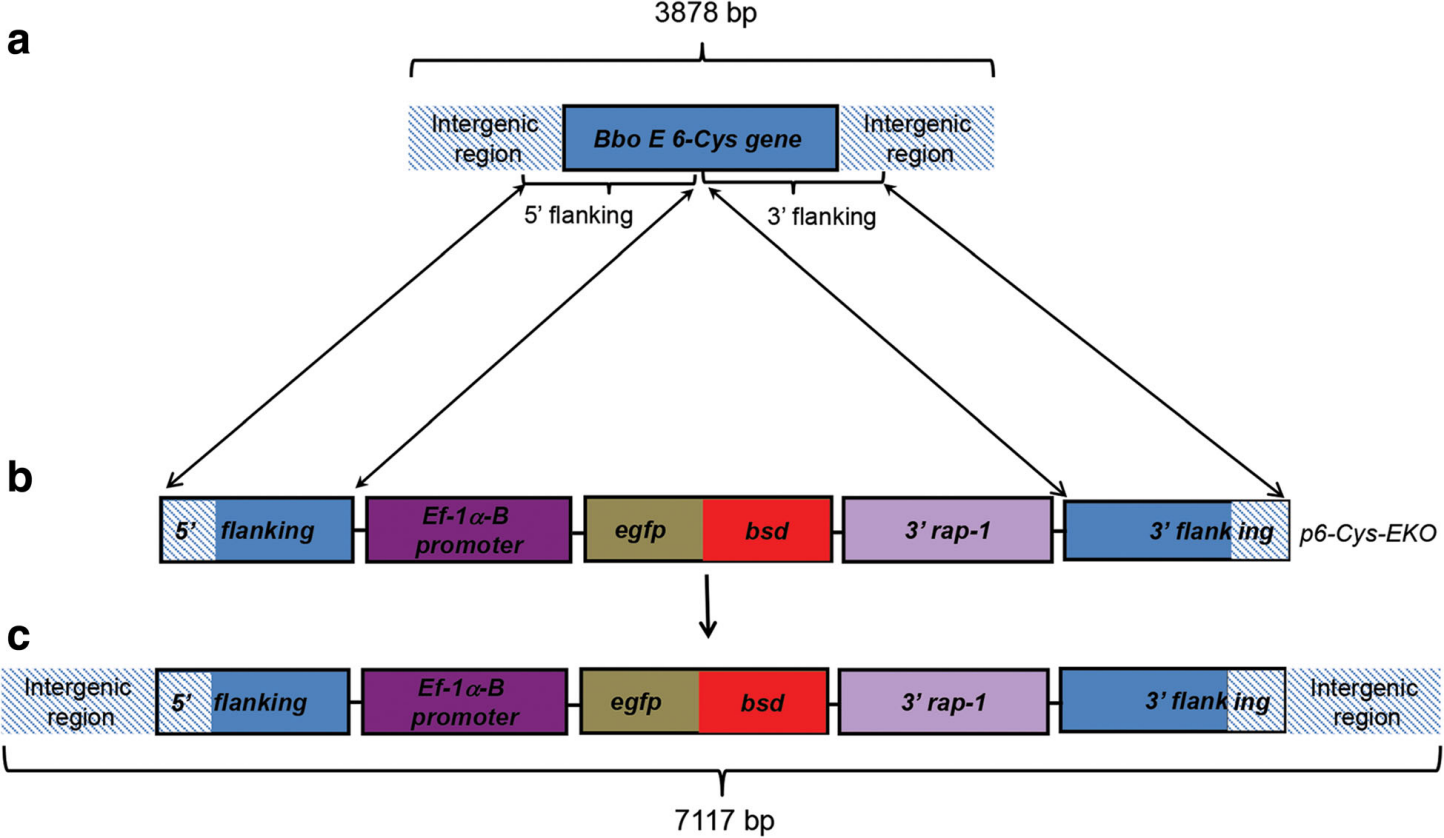
Schematic representation of (**a**) the structure of the 6-Cys *E* gene locus, (**b**) transfection plasmid *p6-Cys-EKO* (GenBank KX247384) and, (**c**) 6 Cys *E* knock out gene

The *B. bovis E* 6-Cys KO plasmid (*p6-Cys-EKO,* Fig. 1) was designed to express the egfp-bsd fusion protein under the control of the *ef-1α* promoter. The *egfp-bsd* gene was amplified by PCR from plasmid *egfp-bsd-pUC* 57 plasmid containing a synthetic *egfp-bsd* fused gene (GenScript, Nanjing, China), using primers *EGFP-F-EcoRI* (5′-GCT ACT **GAA TTC** ATG GTG AGC AAG GGC GAG-3′) and *Tracer-Bsd-EcoR*I (5′*-*TAA TGT **GAA TTC** GCC CTC CCA CAC ATA ACC AGA G-3′). Both primers contain *EcoR*I restriction sites (bold fonts) to facilitate cloning into plasmid *p40-15-luc* [15]. Both, plasmid *p40-15-luc* and the PCR amplified fragment were digested with *EcoR*I. Restriction digestion of *p40-15-luc* with *EcoR*I results in the removal of the luciferase gene. The *EcoR*I-digested PCR fragment was then cloned into the *EcoR*I-treated plasmid *p40-15-luc*. The resulting plasmid, containing the *ef-1α* promoter (*Hindi* III cloning site) now controlling the expression of the *egfp-bsd* gene (*EcoR*I cloning site), and the 3′- region of the *rap-1* gene (3′*-rap-1*) (*Pst*I cloning site) [16, 17], was designated *p40-15-egfpbsd*. To construct the *p6-Cys-EKO* plasmid, we first cloned the 5′ flanking region of the *E* 6-Cys gene (fragment 5′ flanking, Fig. 1a, b) containing ~ 200 bp upstream to the gene 6-Cys *E* locus untranslated region (UTR) plus ~ 900 bp from the full open reading frame (orf) of the full 6-Cys *E* gene into the *Xho*I site of plasmid *p40-15-egfpbsd*. The 5′ flanking end was amplified using the forward primer (5′-**GCG TGC CTC GAG** GTA TTT AAC ATT ACA AAC TCC-3′) and the reverse primers (5′- **GCG TGC CTC GAG** CAT TGG CAT GAA AAG G-3′) containing *Xho*I ends (bold font) for cloning into the *p40-15-egfpbsd* plasmid. The resulting plasmid was designated *p40-15-egfpbsd-5′6-cys*. The 3′ 6-Cys *E* insertion region included ~ 870 bp from the 3′ end of the orf of *E* 6-Cys gene with ~ 200 bp downstream of UTR region of *E* 6-Cys gene. This 3′ flanking region was amplified using the forward primer is (5′-**CGC TAT GGA TCC** TTG ATG GTA ATA TGA GGC-3′) and reverse primer (5′- **CGC TAT GGA TCC** CAG GGA TAG ATA ACC GG-3′) containing *BamH*I restriction sites (bold font), and cloned into the *BamH*I site of plasmid *p40-15-egfpbsd-5′6-Cys*. All primers used for amplification of the 6-Cys *E* flanking regions were designed based on genome sequence information available at http://vmp.vetmed.wsu.edu/research/apicomplexan-genomics [18]. The resulting final transfection plasmid was designated *p6-Cys-EKO* plasmid. The *p6-Cys-EKO* plasmid was fully sequenced, and the assembled final sequence was deposited in GenBank (Accession number KX247384). The *p6-Cys-EKO* plasmid was used to transform Top10 *E. coli* competent cells (Invitrogen, Carlsbad, USA), and purified using Qiagen Plasmid Midi Kit (Qiagen, MD, USA) following the manufacturer’s instructions prior to transfection. Plasmid control *pBluescript* (*pBS*) was purified identically and used as a control in the transfection experiments.

### Transfection of *B. bovis*

The plasmid *p6-Cys-EKO* was electroporated into *B. bovis* Tx T3Bo strain infected erythrocytes as previously described [15, 16]. Plasmid *pBluescript* was used in identical transfections as a negative control. The transfected parasites were transferred to an incubator at 37 °C, selected with blasticidin as described previously [16], and monitored on a regular basis for the presence of transfected parasites with a fluorescence microscope at 60× amplification.

### Cloning transfected parasites

Cloning was performed using a fluorescence activated cell sorter with a Clonecyte attachment (FACS Vantage, Becton Dickenson, Immunocytometry Systems, San Jose, CA) as previously described [19]. The cells were sorted depending on the fluorescence expressed from transfected parasites with the *egfp* gene. Single transfected parasites were sorted into three 96 well plates. The cultures were maintained for 3 weeks as described [20] with a daily change of medium. PCR analysis was performed on 1 μl samples obtained from each well to identify parasites using the *rap-1* primer BoFN primer (5′-TCA ACA AGG TAC TCT ATA TGG CTA CC-3′) and BoRN primer (5′-CTA CCG AGC AGA ACC TTC TTC ACC AT-3′) [20]. PCR positive wells were further analysed by PCR to detect a disrupted 6-Cys gene *E* using a forward primer that anneals in the intergenic region upstream of 6-Cys gene *E* IGE for (5′-GCT AAG CAC CTA TTT AGC GTA AC-3′) and a reverse primer *EF-Pr-R-6-HIII* [5′-GAC CAT AAG CTT AGT AAA CGA TAG AAC AGA CTA AG-3′] [15].

### Phenotypic and genotypic analysis of transfected clones

Multiple approaches were used to define the phenotypic and genotypic characteristics and to confirm the integrity and stability of the transfected clones, as described below.

#### Fluorescence microscopy

Emission of fluorescence by transfected parasites was verified using a Zeiss Fluorescence microscope. Wells containing clones of parasites with uniform bright fluorescence (*N* = 62 clones) were selected for further analysis.

#### PCR and DNA sequencing

PCR assays and sequencing were performed on genomic DNA (gDNA) extracted from the: i] Parental non-transfected strain, ii] *B. bovis* 6-Cys *EKO* (mixed transfected strain)*,* and iii] *B. bovis* 6-Cys *EKO-cln* (transfected clones) to verify the integration and disruption of the 6-Cys *E* gene by the *p6-CysEKO* construct. A PCR amplifications aimed at demonstrating integration and disruption of the 6-Cys *E* locus (Fig. 3a, b) were performed using the primers shown in Table 1. Other comparative PCR analysis was designed to detect the disruption of gene 6-Cys *E* using a forward primer representing the genomic intergenic region upstream of gene 6-Cys *E* named *IGE for* (5′-GCT AAG CAC CTA TTT AGC GTA AC-3′) and the reverse primer *EF-Pr-R-6-HIII* (5′-GAC CAT AAG CTT AGT AAA CGA TAG AAC AGA CTA AG-3′**)** (Fig. 3a) [15].

**Table 1:** Primers sequence used in PCR and sequencing

Amplicon #	Primer pair
1	For: 5′-gctaagcacctatttagcgtaac-3′ Rev: 5′-gaccataagcttagtaaacgatagaacagactaag-3′
2	For: 5′-atgaagcgaaatatcgtacacaatacc-3′ Rev:5′-atcgcaaagctttttcgtaaagttgcaataaattatc-3′
3	For: 5′-ctgatcaagctttatatctgagacaacattagtatcg-3′ Rev: 5′-ctacgaggatcctccttt gtgaggttcacg-3′
4	For: 5′-gctactgaattcatggtgagcaagggcgag-3′ Rev: 5′-ctacgaggatcctccttt gtgaggttcacg-3′
5	For: 5′-gctactgaattcatggtgagcaagggcgag-3′ Rev: 5′-atgtcaatagtgatcatcg-3′
6	For: 5-gctactctgcaggatgagatgcgtttataatg-3′ Rev: 5′-gttgcaaaaacatgttatgaag-3′
7	For: 5′-ttgatggtaatatgaggc-3′ Rev: 5′-gttgcaaaaacatgttatgaag-3′

#### Southern blot analysis

The patterns of insertion of the *EKO plasmid* into the *B. bovis* T3Bo gDNA were analysed using hybridization of undigested and *Bgl*II digested gDNA, using DIG-labeled 6-Cys *E 3*′ *end*, *Egfp-bsd*, *Ef-1α* and *ampicillin* probes in Southern blots. The probes were prepared as previously described [16]. The gDNA used in this analysis included. DNA extracted from the non-transfected parental T3Bo strain, 6*-*Cys EKO, and 6-Cys EKO*-cln*.

#### Clonal line genome sequencing

Approximately 7 μg of gDNA were extracted from in vitro cultured *B. bovis* using the DNEasy kit (Qiagen, Hilden, Germany) with special care being taken to avoid shearing of large DNA fragments. The SMRT Bell Template Prep Kit V1.0 was used for library preparation, and DNA was size-selected at > 15 kb using the Blue Pippon (Sage Biosciences, Edmonton, Canada), the resulting library had an average fragment size of ~ 21 kb. Sequencing was performed on the Pacific Biosciences RSII sequencer using MagBead Loading and P6/C chemistry. Sequences were assembled using the Hierarchal Genome Assembly Process 2 (HGAP2), and the resulting assembly was further analysed using CLC Genomics Workbench. Comparison of the re-sequencing data from the transfected clone to that of the T2Bo reference genome was performed using BLAT 2.0 using default settings [21].

### Phenotypic analysis of mutant strain and clone

To identify the possible impact of the gene 6-Cys *E* mutation on the growth of the *B. bovis* parasite, we compared the ability of the 6-Cys EKO strain, 6-Cys EKO*-cln* and wild-type T3Bo strains to grow in in vitro culture. Cultures of each strain were initiated at 0.5% parasitemia in triplicate wells in the presence and absence of blasticidin [16]. Culture medium was replaced and the parasitemia calculated daily for 8 days. Statistical analysis was performed with the Student *t*-test, and the probability value of less than 5% (*P* < 0.05) was considered significant.

### In vitro neutralisation assay

Inhibition of *B. bovis* merozoite invasion of erythrocytes was performed on the Mo7, T3Bo, EKO, and EKO *cln*, strains as previously described by Hines et al. [22]. Briefly, *B. bovis* merozoites were separated from erythrocytes by centrifugation, and approximately 5 × 10^5^ viable merozoites, as determined using 6-carboxyl fluorescein diacetate [23], were used in each antibody neutralisation reaction: rabbit antisera specific for *B. bovis* 6-Cys E-peptides 1 and 2 [22], or pre-immune rabbit sera. Mouse polyclonal sera against a non-*Babesia* sp. protein (sera specific for Operon-associated protein (OpAG) 2 encoded protein of *A. marginal*) [9] was used as negative control, and a previously described *B. bovis* neutralising monoclonal mouse anti-MSA-1 antibody (BABB35A4) was used as positive control [22, 24]. All sera were heat-inactivated for 30 min at 56 °C, diluted 1:1 in culture medium and incubated with the merozoites for 30 min at 4 °C. Mouse antibodies were used at a concentration of 1.5 μg/ ml. After the incubation period, an equal volume of 5% (v/v) bovine erythrocytes in culture medium was added to each well. The plates were incubated at 37 °C in a 5% CO_2_ atmosphere. Parasites were grown in micro-aerophilous stationary phase culture as previously described [11]. Percentages of parasitized erythrocytes (PPE) were determined every 24 h up to 72 h by counting parasites in smears stained with Diff-Quik on an optical microscopic. The assay was performed in triplicate in 96-well plates. The results were analyzed by the one-way ANOVA with 95% confidence level.

## Results and discussion

### Transfection and biological cloning of transfected *B. bovis* parasites

**Fig. 2:**
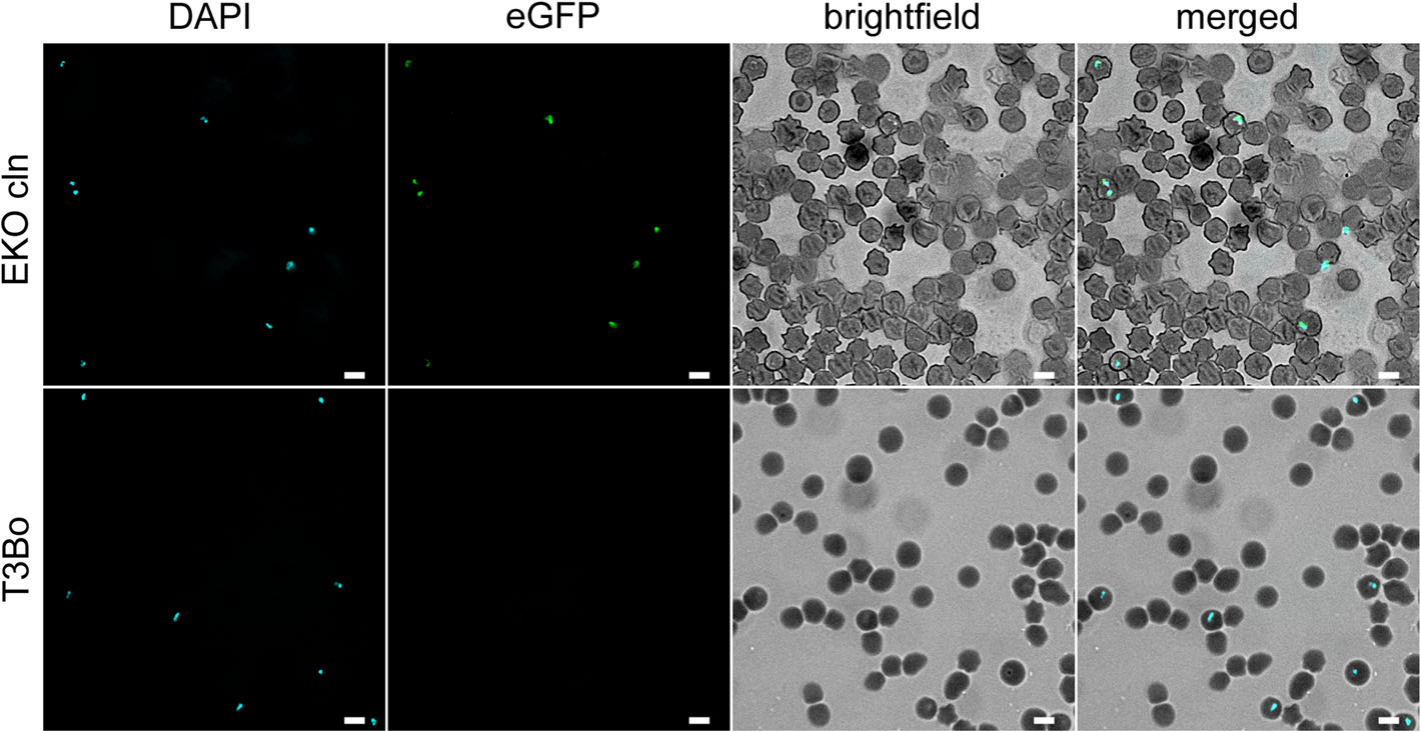
Detection of the expression of *egfp* in *B. bovis* 6-Cys EKO mutant line by fluorescence microscopy. Upper panels: (*left to right*) represents transfected parasites of the EKO *Cln* line stained with DAPI, fluorescent light, brightfield and a merged image respectively. Lower panel identical images obtained using the control non-transfected parental parasite line T3Bo. *Scale-bars*: 5 μm

Parasites of the *B. bovis* T3Bo strain were electroporated with plasmid *p6-Cys-EKO* (GenBank KX247384). Plasmid *p6-Cys-EKO* contains 5′ and 3′ flanking regions designed for disrupting the structure of the 6-Cys gene *E* upon insertion of the *egfp-bsd* selectable marker in the *B. bovis* genome by homologous recombination. Schematic representations of plasmid *p6-Cys-EKO,* the target locus, and the expected structure of the 6-Cys gene *E* in KO parasites, are shown in Fig. 1. Electroporated parasites were selected in in vitro cultures with inhibitory doses of blasticidin [16] starting 4 h after electroporation, and a blasticidin-resistant and egfp fluorescent *B. bovis* line termed *B. bovis* 6-Cys EKO emerged 7 days upon the start of blasticidin selection. In contrast, no parasites transfected with the *pBluescript* plasmid were detected in identically blasticidin-treated cultures at day seven upon the start of selection (Fig. 2). The transfected fluorescent parasite line was grown in in vitro cultures for additional 30 days in the presence of inhibitory concentrations of blasticidin before it was expanded for genotypic analysis and biological cloning. The genotypic analysis of the 6-Cys EKO parasite lines is described below. A clonal transfected *B. bovis* line containing a disrupted 6-Cys gene *E* was then derived from the parasite line *B. bovis* 6-Cys EKO using FACS sorting. FACS sorted single-parasite cells were grown in in vitro cultures developed in a low O_2_ incubator set at 37 °C. The cultured parasites were then screened for the presence of a disrupted 6-Cys gene *E* by PCR. A fluorescent clonal parasite line containing the *egfp-bsd* gene termed *B. bovis* 6-Cys EKO*-cln* emerged in the in vitro cultures 3 weeks after the onset of the FACS cloning. The experiments described below demonstrate the transfected genes effectively disrupt the integrity of the 6-Cys gene *E* in line 6-Cys EKO*-cln.*

### Genotypic analysis of transfected *B. bovis* parasites

**Fig. 3:**
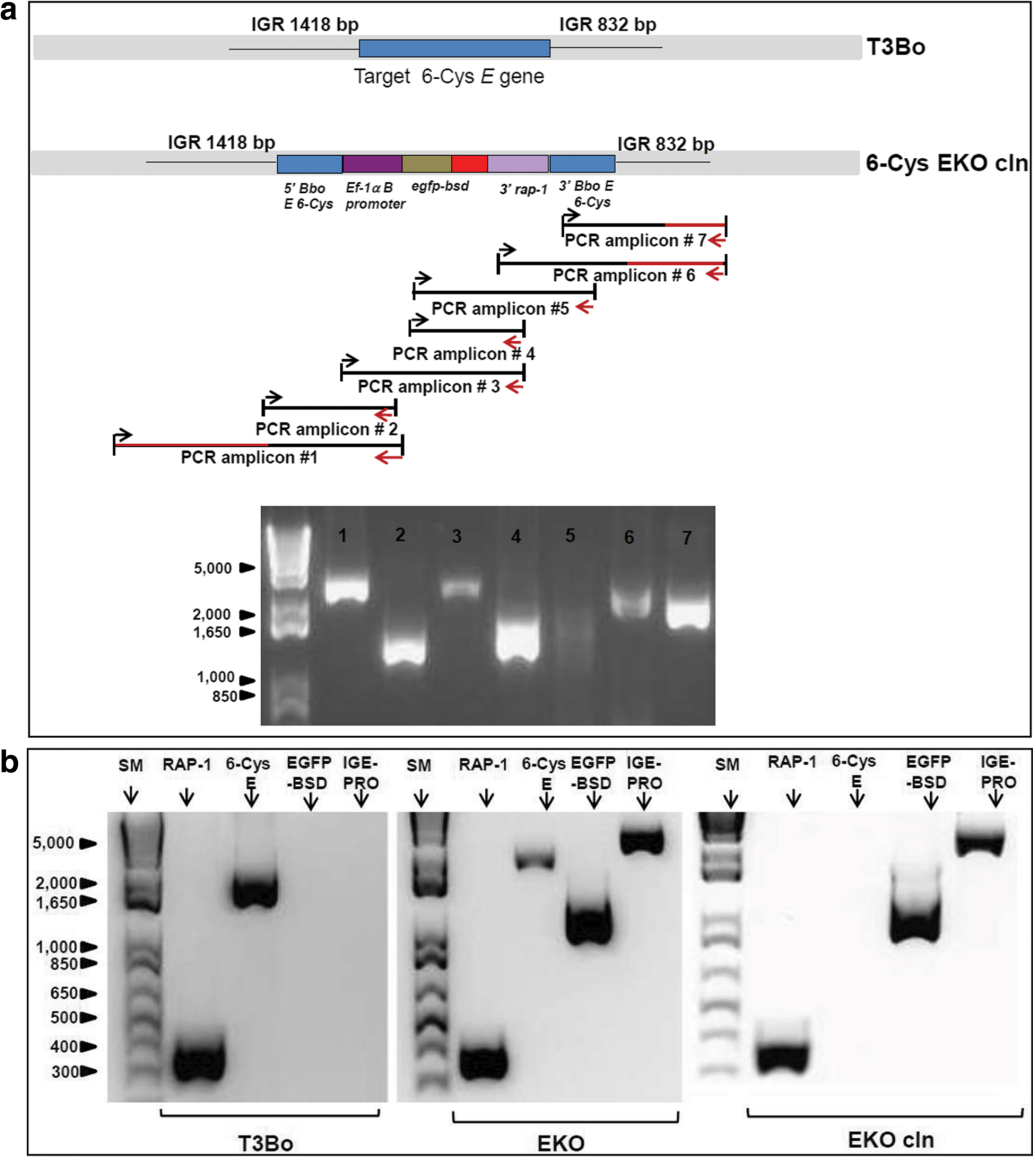
PCR analysis for integration of *egfp-bsd* in transfected *B. bovis*. **a** Top panel: schematic representation of the intact 6-Cys *E* locus in the T3Bo strain and the 6-Cys EKO-*cln* parasites as deduced from full genome sequence of 6-Cys EKO-*cln* parasites. The *red* coloured lines represent gDNA sequences that are not a part of the targeted region included in the transfection plasmid *p6-Cys-EKO*. Lower panel: agarose electrophoresis analysis performed on the PCR products corresponding to each of the fragments numbers 1–7 in the upper panel. **b** Comparative PCR analysis performed on the line EKO-*cln*, T3Bo, and EKO, using RAP-1, 6-Cys *E*, *egfpbsd*, and *IGE*-*Pro* primer sets. SM represents standard molecular weight 10 Kbp

We first performed PCR to characterise the disrupted 6-Cys gene *E* locus in parasites of the line 6-Cys EKO*-cln.* Seven sets of PCR primers were designed to demonstrate disruption of the 6-Cys gene *E* in transfected parasites (Table 1 and Fig. 3a). The results of the seven distinct PCR reactions performed on gDNA extracted from the 6-Cys KO*-cln* are shown in Fig. 3a. Size and sequence comparison of the PCR products are consistent with the disruption of the targeted 6-Cys gene *E* and “in target” insertion of the transfected genes in the 6-Cys EKO*-cln* parasites.

To further confirm specific disruption of the targeted 6-Cys *E* locus, we also performed comparative PCR among the T3Bo, 6-Cys EKO*,* and 6-Cys EKO*-cln* using primers designed for the amplification of *rap-1* (control non-targeted gene), full-size 6-Cys gene *E*, and *egfp-bsd*. Additionally, to demonstrate specific integration at the attempted 6-Cys *E* locus, we also performed a PCR reaction using forward primers IGE (representing sequences in the intergenic region located 5′ of the *E* gene, and not represented in the transfection plasmid *p6-Cys-EKO*) and PRO (representing sequences in the *ef*-1B promoter) as the reverse primer (Table 1 and Fig. 3b). All products derived from these PCR reactions, shown in Fig. 3b were fully sequenced. The control *rap-1* gene was identically amplified from gDNA extracted from all three strains. However, gDNA from strains 6-Cys EKO and 6-Cys EKO*-cln,* but not from the T3Bo strain, generated products when the *egfp-bsd* and *IGE-PRO* primers were employed, confirming the presence of the *egfp-bsd* gene and integration of transfected genes into the 6-Cys gene *E* locus exclusively in the transfected parasites. Furthermore, sequence analysis of PCR amplicons indicates the integration of the structure of the locus by sequences present in the transfected plasmid, which successfully integrated into the genome by homologous recombination. Interestingly, primers designed for the amplification of the full size 6-Cys *E* orf only generated PCR products of identical size and composition in the T3Bo and 6-Cys EKO lines, and no products were visible upon amplification of the 6-Cys EKO*-cln* line, consistent with disruption of the 6-Cys gene *E* in these parasites (Fig. 3b). Taken together, these data also suggest that the transfected line 6-Cys *EKO* contains a mixed population of parasites, either with or without disrupted 6-Cys gene *E*, and that, in contrast, the 6-Cys EKO*-cln* line contains a single insertion of the *egfp-bsd* gene disrupting the integrity of the 6-Cys gene *E* of *B. bovis*.

**Fig. 4:**
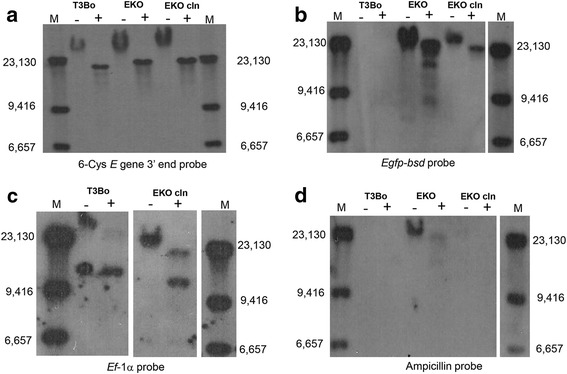
Southern blot analysis of gDNA extracted from non-transfected T3Bo,6-Cys EKO, and 6-CysEKO-*cln*, using Dig-labeled probes against 6-Cys *E* gene 3′ end probe (**a**), *egfp-bsd* probe (**b**), *Ef-α* probe (**c**) and ampicillin probe (**d**). Each sample was analysed using undigested (**-**), or *BglII* digested (+) gDNA. M: molecular marker

Total gDNA extracted from cultured wild-type T3Bo, 6-Cys EKO and 6-Cys EKO*-cln* parasites was then analysed by Southern blot analysis (Fig. 4). The gDNA was digested with *Bgl*II which does not cut inside the *p6-Cys-EKO* plasmid and the 6-Cys *E* locus, separated by agarose gel electrophoresis, and transferred to blotting nylon membranes. The blots were hybridised with dig-labeled specific *egfp-bsd*, *ef-1α*, and ampicillin (plasmid *pBS* marker) probes (Fig. 4a-c, respectively). Taken together, the Southern blot analysis indicated that: (i) both the mixed transfected and clonal strain, but not the wild-type non-transfected, parasites contain a single copy of the *egfp-bsd* gene; (ii) the clonal 6-Cys KO*-cln* hybridize with 6-Cys 3′ probe in a different pattern compared to the wild type and mixed transfected line, suggesting that these parasites contain a disrupted 6-Cys gene *E*; (iii) the ampicillin probe hybridizes with transfection plasmid likely present in the cells as episomal DNA, present only in the mixed line. Plasmid DNA was not detected either in the wild type or the 6-Cys EKO*-cln*; (iv) the patterns of hybridization of the *egfp-bsd* probe suggests that the mixed transfected line 6-Cys *EKO* contains either multiple *egfp-bsd* copies inserted in the genome, or, distinct subpopulations of transfected parasites including non-specific insertions; Yet, the results may also be due to the lasting presence of single or concatenated episomal transfection plasmid (as shown in Fig. 4c) in the transfected parasites of this line; (v) the ef-1α probe hybridized with two bands in the EKO-*cln* line, a ~12 kb band corresponding to the intact *ef-1α* locus, and an upper (~23 kb) band which is identical is size with the fragment hybridizing with the egfp probe (Fig. 4a). These patterns of hybridization were expected and are likely due to the presence of the sequence in the ef-1α probe into the transfection construct that integrated into the *6 Cys* locus.

Collectively, this Southern blot and PCR data support stable insertion of the intended *egfp-bsd* gene disrupting the integrity of the 6-Cys gene *E* orf in in the parasite line 6-Cys EKO*-cln*, as operated by homologous recombination. Full genome sequencing of the 6-Cys *EKO-cln* line again confirmed the gene 6-Cys *E* interruption and is consistent with the PCR and Southern blot data. Sequence analysis of chromosome number 2, where the 6-Cys gene *E* locus is located indicates that the originally 3878 bp targeted area became enlarged to 7117 bp as a result of the integration of the *egfp-bsd* gene, its 3′ rap and the *ef-1α* B promoter regions included in the transfection, interrupting the 6-Cys gene *E*, exactly as described in Figs. 1 and 3a. The pattern of insertion and the conservation of restriction sites derived from the plasmid vector are shown in Additional file 1: Figure S1. The sequence of the interrupted 6-Cys gene *E* region in the 6-Cys E-KO-*cln* parasites was deposited in GenBank (Accession no. KX247383). Analysis of the full genome of the 6-Cys EKO*-cln* gDNA indicated that no other insertions derived from the transfection changes occurred in the genome of parasites of the 6-Cys EKO-*cln* line. Comparison of the original reference *B. bovis* T2Bo genome sequence [18] to the re-sequenced 6-Cys EKO-*cln* revealed random SNP and short insertion/deletion mutations but did not result in gross chromosomal re-arrangements. However, such minor differences among these genomes are expected as they result from comparing sequences derived from a clonal parasite line with a parasite line composed of multiple parasite subpopulations.

### Phenotypic characterization of *B. bovis* transfected parasites

**Fig. 5:**
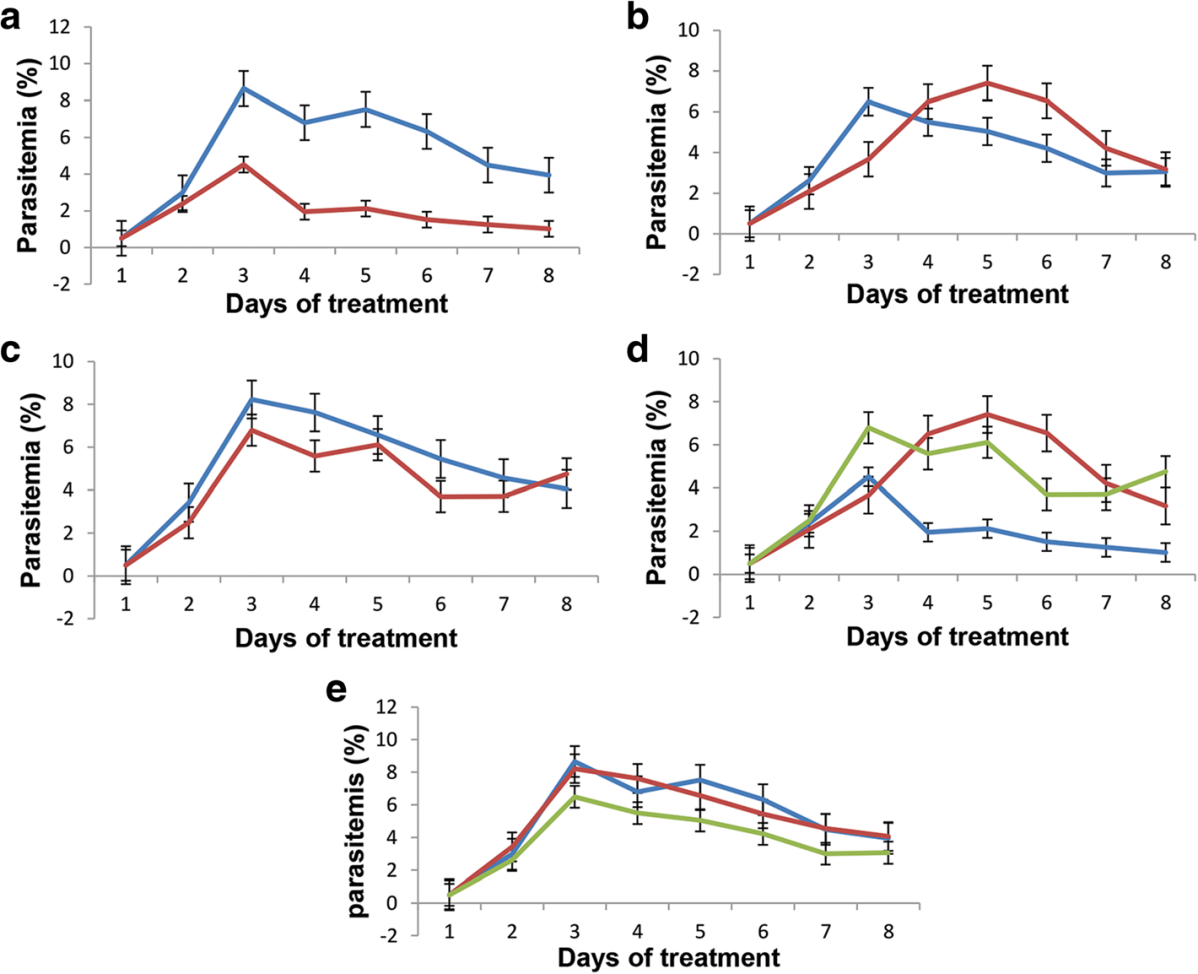
Comparative in vitro culture growth curves of *B. bovis* T3Bo, transfected 6-Cys EKO, and 6-Cys EKO-*cln* parasite lines with and without addition of inhibitory doses of blasticidin. The data are expressed as arithmetic means ± standard deviation (SD). *P* < 0.05 indicates a statistically significant difference and *P* > 0.05 indicating a statistically no significant difference. **a** In vitro culture growth curves of T3Bo strain without (*blue line*) and with the addition of blasticidin (*red line*). (*t*_(12)_ = 4.1, *P* = 0.0013). **b** In vitro culture growth curves of the 6-Cys EKO without (*blue line*), and with blasticidin addition (*red line*) (*t*_(12)_ = -0.55, *P* = 0.58). **c** In vitro culture growth curves of 6-Cys EKO-*cln* line without (*blue line*) or with blasticidin addition (*red line*) (*t*_(12)_ = 0.87, *P* = 0.399). **d** In vitro culture growth curves of T3Bo (*blue line*), 6-Cys EKO strain (*red line*), and 6-Cys EKO-*cln* line (*green line*) with blasticidin addition. Statistical comparisons: T3Bo *vs* 6-Cys EKO lines (*t*_(12)_ = 3.1, *P* = 0.009); T3Bo *vs* 6-Cys EKO-*cln* line (*t*_(12)_ = 3.6, *P* = 0.003). **e** In vitro culture growth curves of T3Bo strain (*blue line*), 6-Cys EKO (*red line*) and 6-Cys EKO-*cln* lines (*green line*) without blasticidin addition. Statistical comparisons: T3Bo *vs* 6-Cys EKO lines (*t*_(12)_ = -1.6, *P* = 0.13); T3Bo *vs* 6-Cys EKO-*cln* line (*t*_(12)_ = -0.25, *P* = 0.80)

The patterns and rates of in vitro growth among the wild type T3Bo, 6-Cys EKO*,* and 6-Cys EKO*-cln* strains were compared. No significant differences (*P* < 0.05) were found in the rate of the in vitro growth among these three parasite lines, regardless of the presence or absence of the selective marker blasticidin in the culture medium (Fig. 5). We concluded from these experiments that a mutation affecting the expression of the 6-Cys *E* gene in the line 6-Cys EKO*-cln* does not affect the fitness of the erythrocyte stages of *B. bovis* parasites while developing in in vitro cultures. In addition, the data demonstrate that the 6-Cys EKO-*cln* parasites can develop similarly in in vitro cultures regardless of the presence or absence of the selective pressure provided by blasticidin. These results suggest that expression of the 6-Cys gene *E* in erythrocyte stages is not essential for the development of *B. bovis* merozoites in in vitro cultures, and suggest that this protein is no longer a viable target for blood-stage vaccine development. However, because it is expressed in the tick stages of the tick, the 6-Cys E protein remains as a potential candidate for the development of transmission-blocking vaccines.

**Fig. 6:**
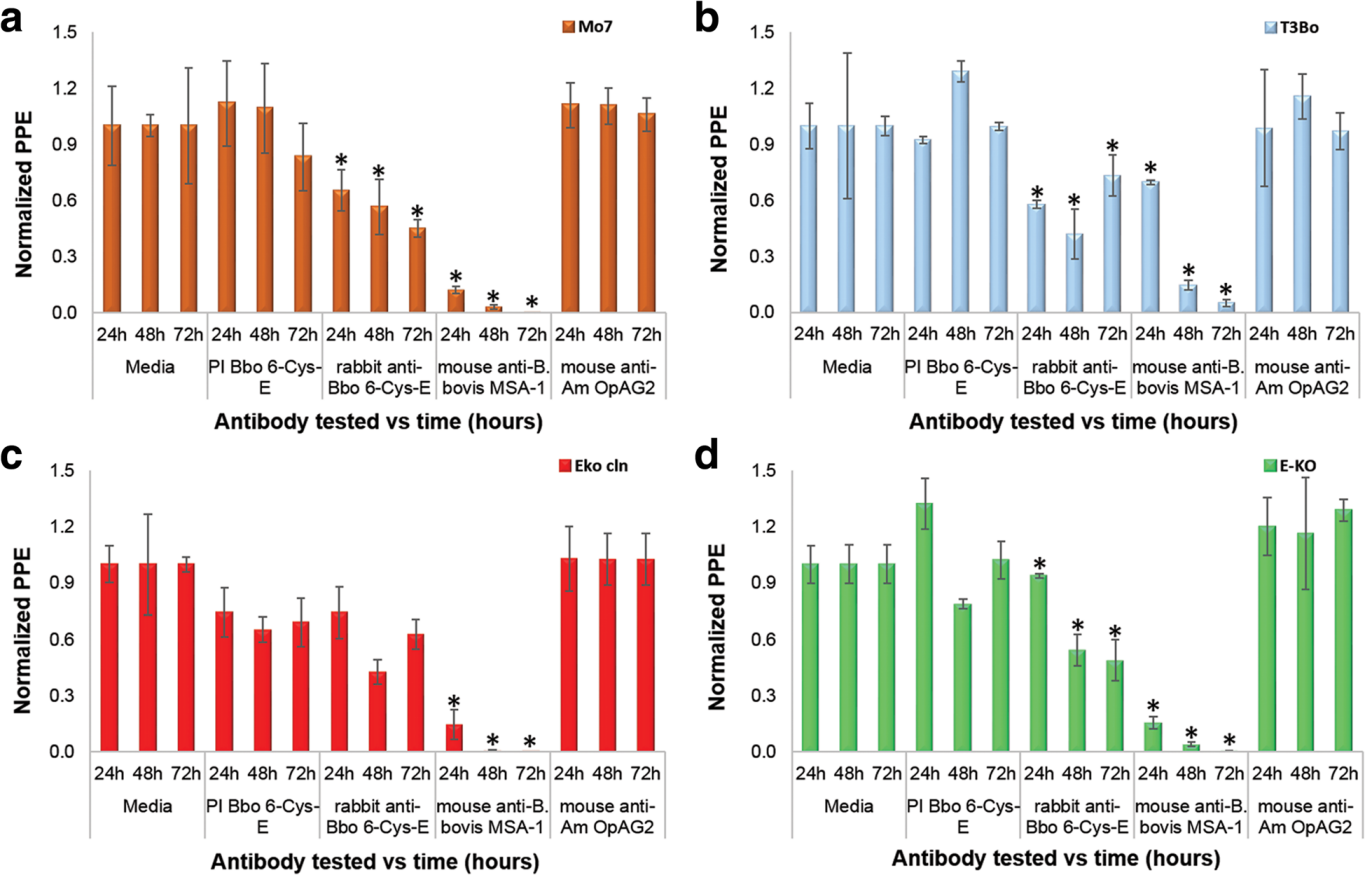
In vitro neutralisation assay performed on Mo7 strain (**a**), T3Bo strain (**b**), EKO- *cln* (**c**), and EKO (**d**). Normalised PPE values (Y axis) obtained from *B. bovis* culture at 72 h in the presence or absence of different sera as indicated on the X-axis. Error bars indicate standard deviations for each sample tested from triplicate culture. Data from each *B. bovis* strain tested, and data from each treatment (pre-immune Bbo 6-Cys *E* and rabbit anti-Bbo 6-Cys-*E*) were compared using ANOVA analysis. (*) represent *P* < 0.05 indicating a statistically significant difference between groups

Previous studies demonstrated surface expression of the 6-Cys gene *E* in the clonal line Mo7. However, identical Western blot analysis performed on T3Bo and 6-Cys EKO parasites using rabbit antibodies against synthetic peptides representing B-cell epitopes in 6-Cys protein E failed in reacting with any parasite product in the T3Bo and 6-Cys EKO parasites (data not shown). In addition, we previously demonstrated that rabbit antibodies directed against synthetic peptides derived from 6-Cys gene *E* [9] have an inhibitory effect on the growth of *B. bovis* Mo7 parasites, suggesting the functional relevance of this protein in erythrocyte stages. An identical in vitro blocking assay [9] was performed in this study on parasites of the Mo7, T3Bo, 6-Cys EKO and 6-Cys EKO*-cln* lines using rabbit pre-immune serum, rabbit anti 6-Cys *E*, monoclonal anti-*B. bovis* MSA-1, and non-related *B. bovis* monoclonal antibodies (*A. marginale* OpAG2). The results of the experiment are shown in Fig. 6, and ANOVA statistical analysis in Additional file 2: Table S1. As expected, the control anti-MSA-1 monoclonal antibodies almost completely abrogate the growth of the parasites at 72 h in all *B. bovis* strains tested. Also, and fully consistent with previously reported data, the rabbit antibodies against the 6-Cys *E* protein [9] are able to inhibit the growth of Mo7 and T3Bo parasites when compared to the pre-immune rabbit sera (*F*_(2,6)_ = 20.55, *P* = 0.002; *F*_(2,6)_ = 6.83, *P* = 0.028, for Mo7 and T3Bo, respectively) (Additional file 2: Table S1) 72 h after the addition of the antibodies, although the levels of inhibition are much lower compared to anti-MSA-1 antibody. However, and consistent with the lack of the 6-Cys *E* gene in these parasites, these antibodies have negligible inhibitory activity on the 6-Cys EKO-*cln* parasites (*F*_(2,6)_ = 4.90, *P* = 0.06) (Additional file 2: Table S1) and a significant inhibitory effect on the growth of the 6-Cys EKO line (*F*_(2,6)_ = 37.02, *P* = 0.000) (Fig. 6 and Additional file 2: Table S1). Marginal inhibition of the 6-Cys EKO line might be due to the presence of *B. bovis* transfected subpopulations in this parasite line that are resistant to blasticidin, but lack a mutation in the 6-Cys *E* gene. Thus, overall, these observations also suggest that, at least under the culture conditions tested in this study, the level of expression of the 6-Cys gene *E* in blood stages is likely decreased compared to the T3Bo parasite line, in contrast to the Mo7 clonal parasite line. It can be hypothesized that the low levels of wild-type *B. bovis* inhibition using anti 6-Cys E antibodies, compared to almost full inhibition caused by the MSA-1 antibody observed in Mo7, and T3B parasites might be due either to low levels or total lack of expression of the 6-Cys *E* in a significant subpopulation of parasites growing in in vitro cultures. This interpretation is consistent with the low levels of 6-Cys *E*-transcripts detected in RNA seq experiments [25]. Moreover, the in vitro neutralisation results are consistent with the lack of expression of the 6-Cys gene *E* in the EKO-*cln*, and thus this clonal line should be suitable for assessing the role of the *B. bovis* 6-Cys gene *E* in the transmission of the parasite by ticks in future experiments. In summary, sequence analysis of the genome of the EKO-*cln* demonstrates effective disruption of the 6 Cys *E* gene in this line. Therefore, it is doubtful that EKO-*cln* parasites can express a full-size version of the protein. However, the possibility remains for expression of a truncated 6-Cys *E* protein, but this is unlikely since the necessary 3′ canonical signals required for RNA processing (such as the poly-A tail) and translation are no longer present in the truncated gene. Another conclusion derived from the phenotypic characteristics of the 6-Cys EKO*-cln* line is that the partial growth inhibition observed in the Mo7 parasites must be likely due to steric hindrance caused by the binding of antibodies to a surface exposed antigen, rather than to a functional requirement for the 6-Cys E protein for the growth of blood stage parasites. This possibility is supported by the observation that parasites unable to express the 6-Cys *E* gene are still able to grow at a similar rate as 6-Cys gene *E* intact parasites in in vitro cultures. Future experiments will be aimed at testing whether 6-Cys *E* mutated parasites can complete the parasite life cycle inside its tick vector.

## Conclusions

In the current study, we were able to produce the clonal line 6-Cys EKO *cln*, and demonstrate that this clonal line contain a disruption of the *B. bovis* 6-Cys gene *E.* The *egfp-bsd* fusion gene insertion in the clonal line 6-Cys EKO *cln* seem to be limited to the intended 6-Cys gene *E*, and transfection does not appear to have altered the genome of 6-Cys EKO *cln* at sites other than that the targeted 6-Cys *E* gene, nor causing any gross chromosomal re-arrangements. The 6-Cys EKO *cln* mutant line has neither obvious morphological nor significant phenotypical differences compared to the parental non-transfected strain T3Bo parasites, and it can grow at identical rates regardless of the presence or absence of the selection agent blasticidin. Moreover, 6-Cys EKO *cln* can resist the blocking inhibitory effects of antibodies against 6-Cys E. Taken together, the data indicate a negligible role of the 6-Cys E protein for the development of *B. bovis* parasite in in vitro cultures. We plan next to compare transmission fitness of the 6-Cys EKO *cln* and 6-Cys intact parasites to start assessing the possible role of the 6-Cys *E* in the transmission of *B. bovis.*

## Additional files

Additional file 1: Figure S1. Simplified restriction enzyme site map of transfection plasmid *p6-Cys-EKO* and in the disrupted 6-Cys locus of 6-Cys EKO-*cln* line, upon integration through homologous recombination. The map was deduced from full plasmid and genome sequencing of *p6-Cys-EKO* and 6-Cys EKO-*cln* parasites, respectively. (TIF 211 kb)

Additional file 2: Table 2. Statistical analysis of the in vitro neutralisation assay for the four *B. bovis* strains tested at 72 h. a Represents the Mo7 strain, b the T3Bo strain, c the EKO-c*ln* line; and, d the EKO line. Sig. represents statistical significance at *P < 0.05. (DOCX 17 kb)
