# Supplementary figures and images for: Geno- and phenotypic characteristics of a transfected Babesia bovis 6-Cys-E knockout clonal line
Source: Parasit Vectors. 2017 May 2;10:214. doi: 10.1186/s13071-017-2143-3 (PMC5414359; doi:10.1186/s13071-017-2143-3)

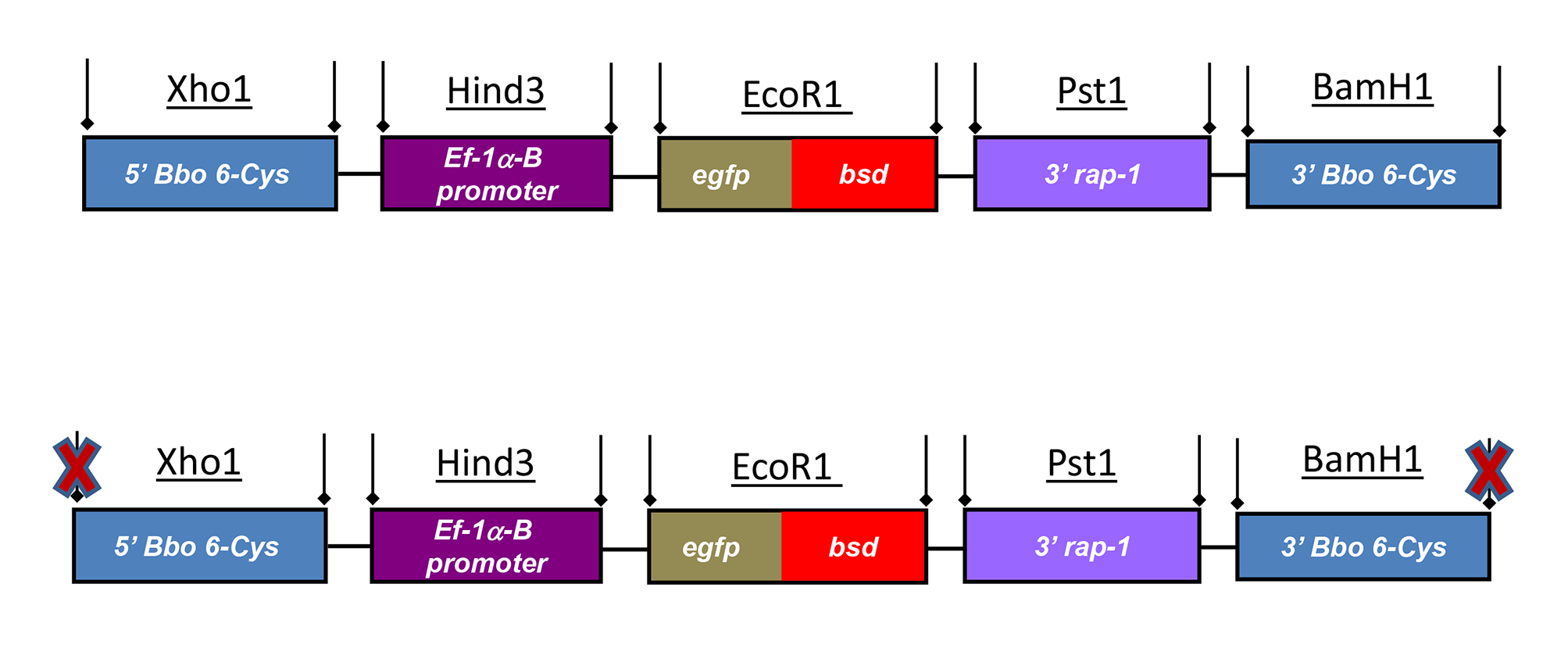

Supplement: Supplementary file 1 — Simplified restriction enzyme site map of transfection plasmid p6-Cys-EKO and in the disrupted 6-Cys locus of 6-Cys EKO-cln line, upon integration through homologous recombination. The map was deduced from full plasmid and genome sequencing of p6-Cys-EKO and 6-Cys EKO-cln parasites, respectively. (TIF 211 kb) [file 13071_2017_2143_MOESM1_ESM.tif]
