# Supplementary material for: Geno- and phenotypic characteristics of a transfected Babesia bovis 6-Cys-E knockout clonal line
Source: Parasit Vectors. 2017 May 2;10:214. doi: 10.1186/s13071-017-2143-3 (PMC5414359; doi:10.1186/s13071-017-2143-3)
Supplement: Supplementary file 2 — Statistical analysis of the in vitro neutralisation assay for the four B. bovis strains tested at 72 h. a Represents the Mo7 strain, b the T3Bo strain, c the EKO-cln line; and, d the EKO line. Sig. represents statistical significance at *P < 0.05. (DOCX 17 kb) [file 13071_2017_2143_MOESM2_ESM.docx]

**Supplementary Table 1:** Statistical analysis of the *in vitro* neutralization assay using anti 6-CysE antibodies at 72 h. Statistical significance *P*<0.05.

***B*. *bovis* T3Bo strain**

|  | **PI Bbo 6-Cys-E** | **Rabbit anti-Bbo 6-Cys-E** |
| --- | --- | --- |
| **PI Bbo 6-Cys-E** | **NS** | **S** |
| **Rabbit anti-Bbo 6-Cys-E** | **S** | **NS** |

S means *P*<0.05. *F*_(2,6)_ = 6.83, *P* = 0.028

***B*. *bovis* 6-Cys EKO line** **at 72h**

|  | **PI Bbo 6-Cys-E** | **Rabbit anti-Bbo 6-Cys-E** |
| --- | --- | --- |
| **PI Bbo 6-Cys-E** | NS | S |
| **Rabbit anti-Bbo 6-Cys-E** | S | NS |

S means *P*<0.05. *F*_(2,6)_ = 37.02, *P* = 0.000

***B*. *bovis* Mo7 strain at 72h**

|  | **PI Bbo 6-Cys-E** | **Rabbit anti-Bbo 6-Cys-E** |
| --- | --- | --- |
| **PI Bbo 6-Cys-E** | NS | S |
| **Rabbit anti-Bbo 6-Cys-E** | S | NS |

S means *P*<0.05. *F*_(2,6)_ = 20.55, *P* = 0.002

***B*. *bovis* 6-Cys EKO-*cln* line at 72h**

|  | **PI Bbo 6-Cys-E** | **Rabbit anti-Bbo 6-Cys-E** |
| --- | --- | --- |
| **PI Bbo 6-Cys-E** | NS | NS |
| **Rabbit anti-Bbo 6-Cys-E** | NS | NS |

NS means *P*>0.05. *F*_(2,6)_ = 4.90, *P* = 0.06
